# Monitoring patients on chronic hemodialysis and post renal transplant for chronic viral infections

**DOI:** 10.1186/1471-2334-13-S1-P68

**Published:** 2013-12-16

**Authors:** Adriana Bold, Gabriela Iliescu, Camelia Piciu, Vladu Iulia

**Affiliations:** 1 University of Medicine and Pharmacy Craiova, Romania; 2 Emergency Clinical County Hospital Craiova, Romania

## Background

Patients receiving hemodialysis may be at increased risk for acquiring an infection with hepatitis B or C virus (HBV or HCV). Different reports show high prevalence of chronic viral infection among chronic dialysis patients, involving a distinct clinical problem with many challenging issues: the immunosuppressive effect of renal failure, the susceptibility for various infections, the long-term implications on morbidity and mortality and the option for kidney transplantation.

## Methods

We conducted a study on a series of forty current chronic dialysis patients and on four patients in the first year after a renal transplant. Tests were performed on Cobas e411, ECLIA assay for HBsAg and anti-HCV.

## Results

Dialyzed patients were 23 men and 17 women out of which 16 (40%) were positive for one viral infection: 9 (56.2%) for HBsAg and 7 (43.8%) for anti-HCV. Patients in the anti-HCV positive group were more likely to be older (ages 55.4±10.0 versus 48.8±13.2 years old) and men (7 vs. 5). Positive cases of HBsAg were equal between men and women. All patients had similar risk of death. Transplanted patients, ages between 30 and 40 years old: three men with positive HBsAg and one woman co-infected: with HBV in the dialysis period, and with HCV post-transplant. She was at increased risk for death and had a lower quality of life compared with the monoinfected men and died 2 years after the transplant.

## Conclusion

Chronic viral HBV and HCV infection increased the risk for death and impaired the quality of life among dialysis and renal transplant patients. Also with multiple comorbidities, they need special care from clinicians. Further studies should assess the measures used to prevent and treat HBV and HCV infection.

